# Tobacco control in Nigeria- policy recommendations

**DOI:** 10.1186/1617-9625-10-8

**Published:** 2012-06-19

**Authors:** Israel Agaku, Adisa Akinyele, Akinbode Oluwafemi

**Affiliations:** 1 Africa Tobacco Control Regional Initiative (ATCRI), Plot 397B, George Crescent, Agbalajobi Estate, off Wempco Road, Lagos, Nigeria; 2 Department of Oral Pathology, University College Hospital (UCH) Ibadan, Ibadan, Nigeria

**Keywords:** Policy, Cigarettes, Tobacco, Smokeless, Bans, Product appeal

## Abstract

Major strides towards national tobacco control have been made since Nigeria became signatory to the WHO Framework Convention on Tobacco Control (FCTC) in June 2004. The Nigerian senate passed a bill on March 15, 2011 which is expected to be signed into law shortly, to regulate and control production, manufacture, sale, advertising, promotion and sponsorship of tobacco or tobacco products. This paper highlights how the proposed tobacco control law provides a unique opportunity to domesticate the WHO FCTC, expand on smokeless tobacco regulation and develop a science base to improve tobacco control measures in Nigeria.

## Letter to the editor

On March 15, 2011, the Nigerian Senate passed a bill which is expected to be signed into law shortly, to regulate and control production, manufacture, sale, advertising, promotion and sponsorship of tobacco or tobacco products [1]. This proposed law represents a positive step towards addressing the problem of tobacco in Nigeria and reducing all forms of tobacco related disease. It also provides a unique opportunity to domesticate the WHO Framework Convention on Tobacco Control (FCTC). The essential components of the bill include; A National Tobacco Control Committee to guide implementation and future tobacco control policies; a comprehensive ban of smoking in public places; clearly visible tax stamps on cigarette packs; bans on sales to minors and by minors; a comprehensive ban on advertising, sponsorship, and promotion; and health warnings covering 50 percent of the display area of tobacco packages with the Ministry of health empowered to prescribe pictures or pictograms and enforce provisions.

A significant policy gap though is the failure of the bill to address the problem of smokeless tobacco. Experience from developed countries has shown that policies specific to smokeless tobacco must be developed and implemented to ensure successful tobacco control. The Euromonitor has predicted a 7% growth in volume for cigarette from 2010–2015 and an astounding 77% growth for smokeless tobacco for Nigeria [2,3].

**Figure 1:**
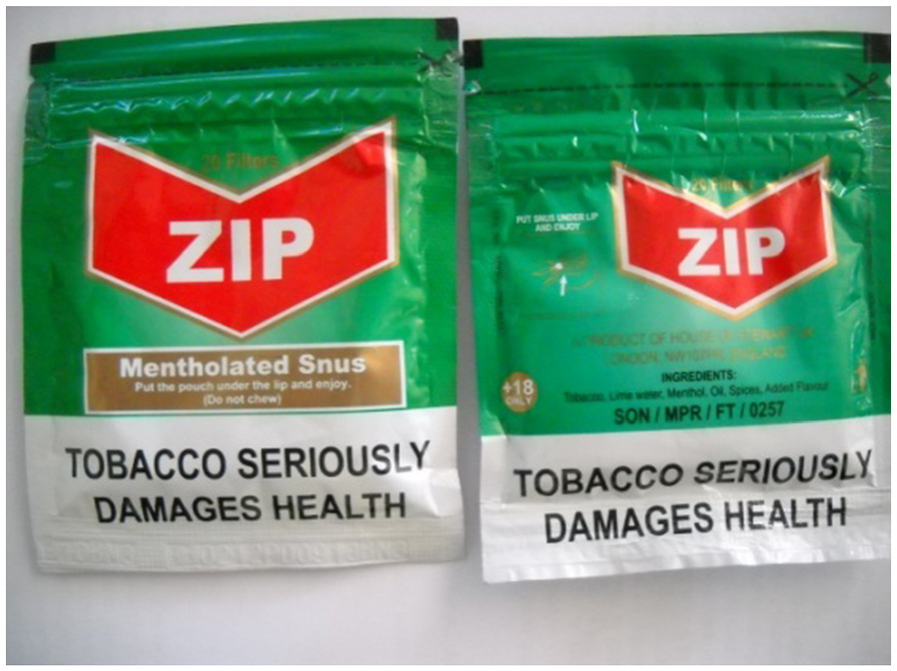
Recently introduced packaged and mentholated smokeless tobacco product in Nigeria.

**Figure 2:**
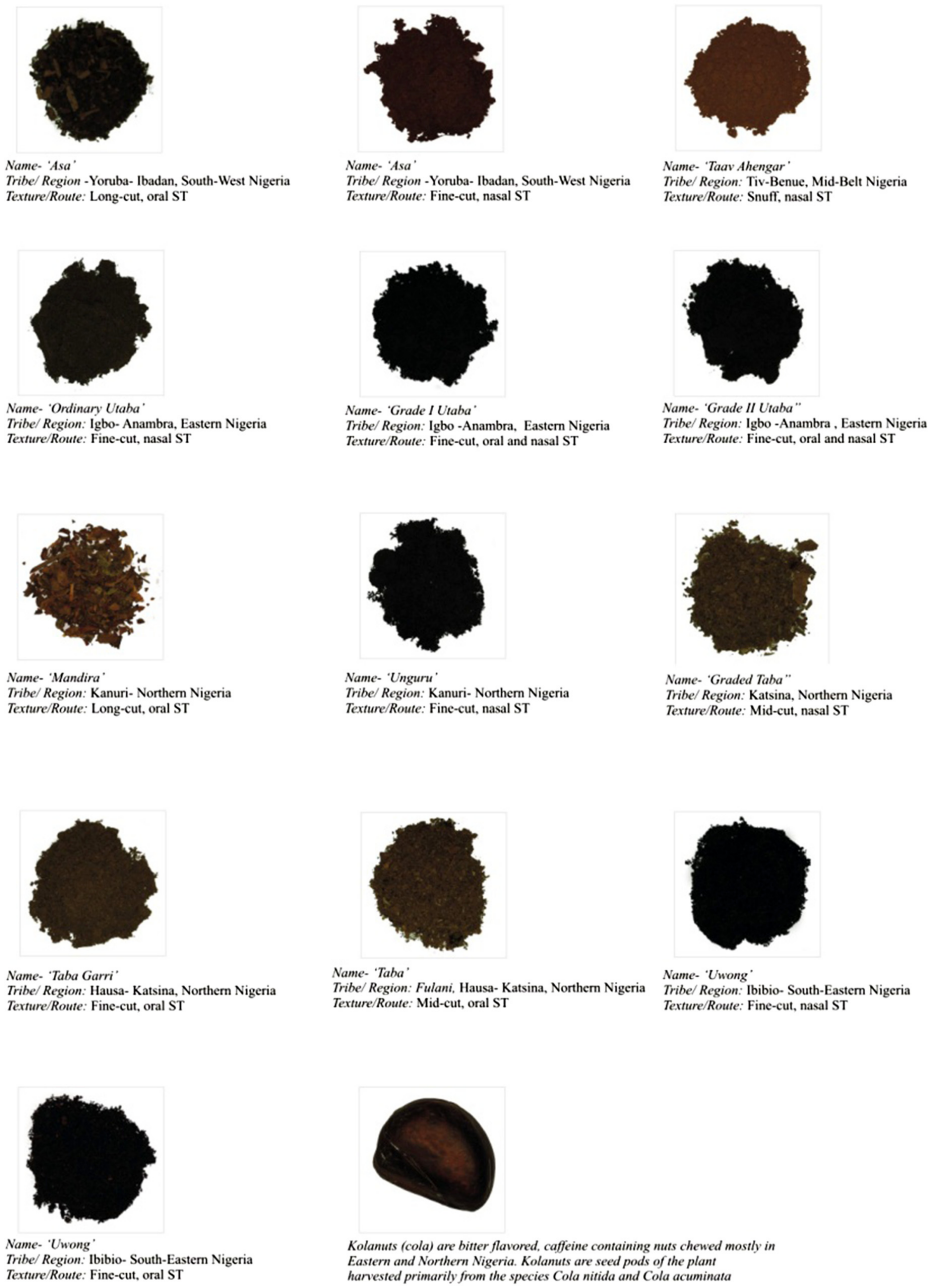
Traditional Smokeless Tobacco Products used in the major Geo-ethnic Regions in Nigeria.

Smokeless tobacco marketing and use in Nigeria has evolved significantly in the past few years. A major development in 2010 was the introduction of ‘Zip’; a Swedish-style snus by a JTI subsidiary- West African Tobacco (Figure 1).This is now marketed in two variants: the standard Zip and the mentholated Zip Cool which aims to tap into the preference in Nigeria for mentholated tobacco products. Its sales in 2010 were estimated at 12 tons valued at NGN 0.1 billion (approx. $634,328) [3]. Distribution of smokeless tobacco products is still in a nascent stage and is mostly through independent small grocers, which are largely kiosks that sell a range of food, drinks, and tobacco items, and has not widened to include other channels such as supermarkets. These new smokeless products are significantly different from the traditional smokeless tobaccos in Nigeria both in packaging and overall product appeal (Figure 2). These developments highly underscore the need for SLT regulation in Nigeria. Successful tobacco control measures regarding smokeless tobacco will create an environment for success in controlling the emerging smokeless tobacco epidemic in other parts of Africa.

## Cigarette pricing and warning labels in Nigeria

**Figure 3:**
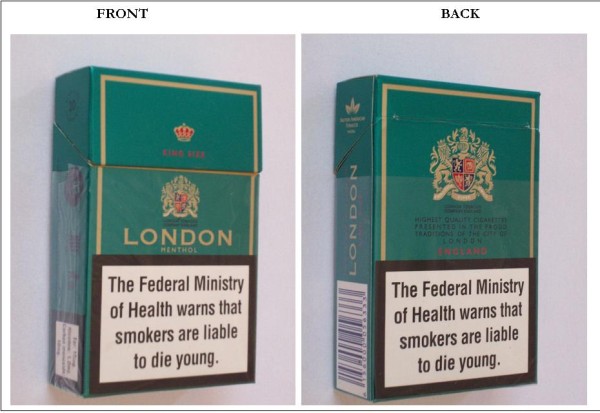
Front and Back views of Cigarette pack in Nigeria showing text-only warning labels.

As of June 2011, the price of the lowest priced 20 cigarette-pack in Nigeria was about NGN50 (approx. $0.32) while premium cigarettes were sold for about NGN200 (approx. $1.27). Increasing retail cigarette prices in Nigeria may decrease smoking and smoking-related disease, particularly among children who are price sensitive, and would also generate revenue for the government. Cigarette packs in Nigeria currently contain text-only warnings; “The Federal Ministry of Health warns that smokers are liable to die young”, which covers approximately 30% of the front and 40% of the back (Figure 3). Domestication of Article 11 of the WHO FCTC regarding pictorial warning labels may result in a reduced prevalence of youth smoking in Nigeria. Pictorial warnings, when used appropriately evoke negative emotive feelings of fear and disgust, and are readily understood by a diverse audience regardless of age or secular education [4]. Graphic package warning labels comprised of a picture embedded with a text message relevant to the picture can be integrated with larger interventions such as mass media campaigns to educate smokers and prevent smoking initiation [5].

## Monitoring the impact of the proposed tobacco control law- areas for further policy action

For the tobacco control law to be realized to its fullest extent, it is critical to include surveillance systems to monitor compliance with the bans on cigarette use and advertising as well as develop policies and interventions for smokeless tobacco use. The addictiveness and toxicity of the new SLT products in Nigeria need to be determined and compared with products sold in developed nations. There is a need for a nationally representative case–control study to demonstrate and measure the association between smokeless tobacco use and oral diseases including oral and nasal cancer in Nigeria.

To measure the effectiveness of the proposed law, longitudinal studies need to be conducted to evaluate compliance with restrictions in public smoking and advertisement ban. Longitudinal measurement of secondhand smoke exposure using P.M _2.5_ levels in public areas such as hospitals, schools and hospitality centers such as bars and clubs will be instrumental in assessing the impact of the law in reducing secondhand smoke. Economic research is also indicated to estimate the health-related cost of smoking in Nigeria, as well as the impact tobacco control policies may have on the economy.

## Policy recommendations for tobacco control in Nigeria

We recommend specific policy actions to help ensure successful tobacco control in Nigeria. The adoption and implementation of the law must be a collaborative effort between Federal, state and local governments. While the Federal government should assume oversight of the adoption and implementation of the ban, state and local law enforcement agencies have to work collaboratively to ensure that institutions, business (e.g. hospitality venues), and individuals within their jurisdictions are fully compliant with the policy.

Enforcement of the smoking ban

➢ Smoking in public spaces including public buildings (such as hospitals, schools); vehicles (such as trains, buses); childcare facilities or within 20 feet of such designated spaces carries a prohibitive fine of up to N5000 ($30).➢ Failure on the part of owners of hospitality venues to control smoking within their premises carries a fine of up to N20, 000 ($120) for the owner, and up to N5000 ($30) for the primary offender.

➢ All hospitality venues should be required to put up signage at conspicuous sites such as doors and well-lit areas inside and outside of their venues prohibiting smoking. No ashtrays or ashtray equivalents such as cups or candleholders shall be allowed within hospitality venues.

Disruption of acts of violation or attempts at violation of the smoking ban at public places

➢ Sting operations by members of the Law enforcement agencies at the Local, State and Federal level, particularly at nightclubs, to ensure full compliance with the law. Third-time offending proprietors may risk losing their license to operate a hospitality venue as well as a fine of up to N50, 000 ($300).

➢ Toll-free numbers for calls and or text messages should be provided, and conspicuously displayed at all public spaces. Ideal numbers should be easy to remember (e.g. 0803 SMOKER). Members of the public should be able to call or send text messages to law enforcement agents in the instance of a violation of the policy.

Restriction of sales, advertisement and illegal trade

➢ No sale of tobacco products to or by minors under 18. Sale of cigarettes or smokeless tobacco products shall be restricted to adults possessing valid government issued identification such as a driver’s license, National ID card, or voter’s license card.

➢ Prohibit sale of cigarette in single sticks. We further propose increasing the retail price of a standard cigarette pack from N50 ($0.3) to N280 ($1.72).

➢ All point of sale advertisements should be removed. Sponsorship of events by tobacco industry at government owned premises or institutions such as Federal and state universities, Hospitals, and related establishments shall be disallowed.

➢ Enforcement of Pictorial warning labels on cigarette packs, occupying at least 50% of the front and back of the packs.

➢ Smuggling of smokeless and cigarette tobacco products into the Country should be checked. Tighter security measures should be enforced at the border to prevent the massive influx of illegal tobacco products into the country. Offenders who are caught should be punished to the fullest extent of the law.

Monitoring and evaluation

➢ Formation of an independent council/committee, adequately funded, and charged with the responsibility of providing scientific leadership in monitoring and evaluating the law. The Council should represent a cross-section of experts in the areas of epidemiology, biostatistics, medicine, and health policy. Among other research needs, it would be crucial to set up a comprehensive national smoking surveillance to monitor and evaluate geographic and temporal trends in tobacco use nationally.

Ensuring a sustainable tobacco control in Nigeria requires a strong coalition with representatives from various stakeholder groups including government, health professionals, NGOs, media and the lay public. Such collaborations will help domesticate the FCTCs in Nigeria and provide a model for replication in other African countries.

## Competing interests

The authors declared that they have no competing interest.

## Authors’ contributions

Authors AI, AA conceptualized the study, collected and interpreted data; author AO revised the document carefully for important policy content, all authors contributed towards the drafting and final review of the manuscript.
